# Smart Bandage Based on Batteryless NFC for Wireless Pressure and Wound State Monitoring

**DOI:** 10.3390/bios16050300

**Published:** 2026-05-21

**Authors:** Marco Cujilema, Ramon Villarino, David Girbau, Antonio Lazaro

**Affiliations:** Electronics, Electrical and Automatics Engineering Department, Rovira and Virgili University, Av. Paisos Catalans 26, 43007 Tarragona, Spain; marcorodrigo.cujilema@urv.cat (M.C.); ramon.villarino@urv.cat (R.V.); david.girbau@urv.cat (D.G.)

**Keywords:** smart bandage, Near-Field Communications (NFC), wound monitoring, pressure monitoring, energy harvesting, battery-less

## Abstract

Although compression therapy is widely used to improve wound healing, selecting the appropriate pressure remains a challenge in clinical practice. This work proposes an intelligent patch integrated into a bandage that allows for the simultaneous monitoring of the applied pressure and wound condition using Near-Field Communication (NFC). The proposed patch integrates a force-sensitive resistive sensor to measure pressure and a capacitive sensor to detect wound exudate through capacitance variations. Capacitance is obtained by analyzing the delay in the stepwise response of the sensor, while resistance is measured from the voltage drop across a resistive divider, which is read by a microcontroller’s analog-to-digital converter. The system is powered wirelessly through NFC energy harvesting, triggered by a mobile device that acts as a reader. The NFC module can be moved away after measurement to improve patient comfort or remain integrated into the dressing for periodic monitoring. Experimental results demonstrate pressure measurements up to 140 mmHg and exudate detection up to 200 μL, confirming the feasibility of battery-free NFC smart bandages for therapeutic monitoring based on wound compression.

## 1. Introduction

Chronic wounds represent a substantial burden on the global health system. In the U.S., treatment costs exceed $25 billion annually [1], while in Spain, the estimated annual expenditure for chronic wound care within the public primary healthcare system surpasses €1.7 billion [2]. Furthermore, an analysis conducted in five European countries estimated that the direct costs attributable to venous ulcers exceed $10.7 billion annually [3]. Beyond the economic costs, chronic wounds significantly reduce patient quality of life and are associated with recurrent infection, pain, and mobility impairment [4]. Despite this substantial socioeconomic burden, assessment in routine practice still relies primarily on periodic visual inspection. This protocol requires bandage removal, which alters the condition of the wound and consequently delays tissue regeneration. Continuous, non-invasive monitoring systems have not yet been incorporated into routine clinical practice, and most of the devices that make up these systems are still in the research and development phase [5]. Furthermore, repeated dressing removal can negatively affect the moist wound-healing environment and compromise tissue regeneration [6].

Venous leg ulcers (VLUs) are among the most common chronic wounds, with a combined point prevalence of 0.32% and an incidence of 0.17% [7]. Compression therapy remains the gold standard treatment [8], requiring a graduated pressure gradient—typically 40–45 mmHg at the ankle and 15–20 mmHg at the knee—to improve venous return and accelerate healing [9]. However, the interface pressure exerted by a multilayer bandage depends largely on variables such as bandage tension, limb perimeter, and the number of layers applied. These factors often cause an uneven distribution of pressure in different anatomical areas. This pressure variability can compromise therapeutic efficacy unless real-time monitoring mechanisms are implemented. However, in routine clinical practice, accurately monitoring pressure under the bandage remains a challenge, as it depends on the level of compression applied by the healthcare professional [9]. Moreover, wearable sensing systems capable of continuously monitoring bandage remain limited by challenges related to miniaturization, power management, and system integration [10].

In addition to pressure control, the wound’s moisture level is a critical indicator of healing progress and exudate accumulation. Accordingly, a multimodal sensing approach that integrates pressure and moisture monitoring enables a more comprehensive assessment of bandage effectiveness than using a single parameter. Therefore, continuous monitoring of both parameters is essential to optimize compression therapy while preserving tissue integrity.

To address these clinical challenges, several wireless sensing strategies have been investigated in recent years. Pressure sensors based on resonant LC structures have been proposed in the literature, in which pressure variations modify the capacitance and, consequently, shift the resonant frequency [11,12,13,14]. In this context, the authors have published a work using passive resonant architectures based on LC circuits for battery-free monitoring of pressure and humidity [15], demonstrating the feasibility of this approach. However, these systems often demand specialized radio-frequency equipment (e.g., a vector network analyzer) for tracking and reading resonances, which limits portability and clinical deployment. Unlike the LC-based architecture, the system proposed in this work allows for direct interrogation via a smartphone, features embedded digital signal processing through an integrated microcontroller and uses an NFC energy harvesting interface.

From the perspective of materials and system integration, flexible electronics provide better mechanical adaptability by reducing stress and preserving microcirculation during prolonged use [16,17]. This property is critical in the treatment of chronic wounds, where thin-film pressure sensors must not damage the skin around the wound. For this reason, wireless pressure sensors have been developed for continuous medical monitoring [18,19].

Energy-harvesting architectures eliminate onboard batteries, reducing maintenance and increasing lifespan. Recent developments in battery-free wireless sensing platforms have highlighted the potential of wearable sensors and those worn directly on the skin for continuous biomedical monitoring applications [20,21]. Near-Field Communication (NFC), which operates at 13.56 MHz, enables short-range wireless communication and is compatible with smartphones [22]. Modern NFC integrated circuits (ICs) incorporate energy harvesting capabilities, obtaining several milliwatts from the magnetic field generated by the reader, enough to power up low-power microcontrollers and sensors [23]. Unlike UHF RFID or Bluetooth-based systems, NFC operates in the near-field magnetic regime, which is inherently less affected by tissue-induced detuning and therefore provides more stable operation for on-skin and sub-bandage applications [24,25]. Since this architecture enables the direct use of widely available smartphones, it makes it easier to implement the system in outpatient clinics and for home care cases, thereby eliminating the need for specialized radiofrequency equipment and improving scalability and clinical accessibility. Although flexible smart bandages have multiple electronic functions for wound care [26], most multimodal systems still rely on battery-powered architectures, which increase device thickness and maintenance requirements and can affect long-term reliability [27]. These limitations underscore the need for completely passive, low-profile sensing platforms. Existing passive LC sensors allow for battery-free operation but require external RF interrogation hardware, which limits portability [28]. Telemetry-based wearables provide continuous monitoring, but rely on battery-powered electronics and communication modules that increase thickness and maintenance demands [29]. Meanwhile, smart wound dressings that integrate biochemical or temperature sensing do not provide interface pressure monitoring under compression therapy conditions [30]. Existing research allows for the monitoring of wounds using battery-free LC-based dressings [14] and NFC-based sensing platforms [22,23], but these approaches do not allow for the simultaneous measurement of pressure and wound state via a direct interface such as a smartphone.

In this context, this work proposes a battery-free NFC-based sensing platform that simultaneously monitors bandage interface pressure and wound moisture using a smartphone as reader, eliminating the need for laboratory-specific RF instrumentation and onboard batteries. The system consists of a multilayer patch that integrates a pressure-sensitive material connected to interdigital electrodes in the top layer. The bottom layer integrates a parallel-plate capacitor whose capacitance varies according to the dielectric permittivity associated with the moisture level. The applied pressure modifies the effective resistance between the electrodes, while variations in humidity, which depend on the state of the wound, produce changes in the capacitance of the system. Both electrical magnitudes are acquired and processed by an NFC board that includes a microcontroller and an energy harvesting circuit. DC power to feed the electronics is harvested directly from the magnetic field generated by the smartphone. Thus, the proposed architecture offers a compact, battery-free, portable solution for non-invasive monitoring.

The paper is organized as follows. Section 2 presents a comprehensive overview of the challenges associated with adjusting bandage pressure and highlights its importance. Section 3 provides an overview of the system, including the pressure and moisture sensors, as well as their measurement principles. The experimental validation and performance analysis of the proposed sensors are presented in Section 4. Finally, Section 5 summarizes the main conclusions and outlines potential future lines of research.

## 2. Bandage Pressure

The application of controlled pressure through compression therapy is a cornerstone in the treatment of various wounds, including chronic ulcers, burns, and post-surgical lesions [31]. Properly applied bandage pressure supports wound healing by improving blood circulation, reducing fluid accumulation, and stimulating tissue regeneration [32]. Maintaining optimal pressure prevents complications such as ischemia or ineffective therapy. The pressure level varies depending on the type of wound, its location, and patient’s condition, which infers the need for personalized compression therapy [33].

Table 1 classifies bandage pressures in mmHg (1 mmHg = 133 Pa) according to their ranges and summarizes their typical applications. The applied pressure depends on the location. For example, in the treatment of chronic venous ulcers, the optimal pressure profile at the ankle is around 40 to 45 mmHg, while at the knee it decreases to 15 to 20 mmHg [34]. If excessive pressure is applied to the bandage, it can cause tissue damage, pain or necrosis, rendering compression therapy ineffective.

The interface pressure applied by a bandage depends on both the limb radius and the bandage properties. A comprehensive mathematical model to determine the applied interface pressure as a function of the number of bandage layers is presented in [35] based on Laplace’s law. For multilayer bandages, the increase in radius caused by each additional layer must be considered. The thick wall cylinder theory [36] predicts the calculation of the pressure (in Pa) exerted by *N* layers of bandage, also considering the radial stress in the direction of the bandage thickness from the expression [35]:

(1) $$P=\sum_{i=1}^{N}\frac{T_{i}\left(D_{i}+t_{i}\right)}{\frac{1}{2}W\cdot D_{i}^{2}+W\cdot t_{i}\cdot \left(D_{i}+t_{i}\right)}$$

where *D* is the initial diameter of the limb, *i* denotes the bandage layer, $t_{i}$ and *W* are the thickness and width of the *i*-th bandage layer, respectively, and $T_{i}$ is the tension of the bandage (in N) applied during each wrapping turn. $D_{i}$ is the diameter of the limb including the thickness of the previous bandage layers:

(2) $$D_{i}=D+\sum_{i=1}^{N}2t_{i-1}$$

For a thin bandage layer, the expression is simplified based on the prediction derived from thin-walled cylinder theory, as reported in [36]:

(3) $$P=\sum_{i=1}^{N}\frac{2T_{i}}{W\cdot D_{i}}$$

**Figure 1:**
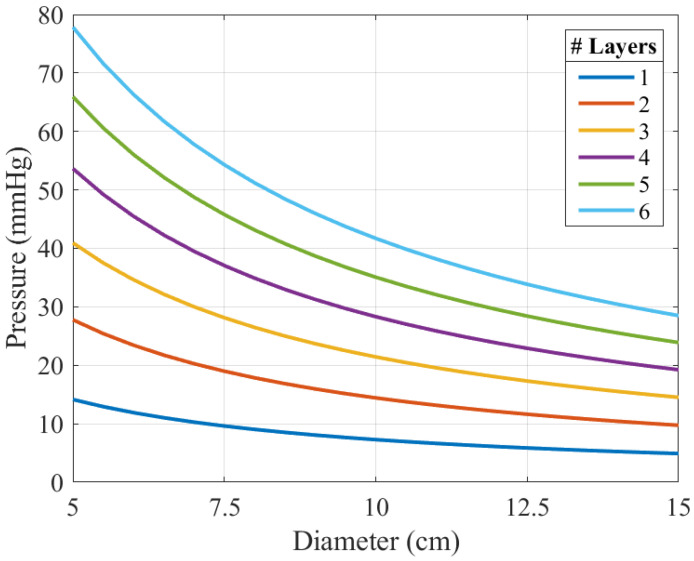
Computed interface pressure as a function of limb diameter D for different numbers of bandage layers, based on the thick-walled cylinder model, assuming a tension of 4 N per turn and a width *W* of 10 cm.

Figure 1 shows the pressure in mmHg calculated using Equation (1), after applying a typical tension of 4 N. This pressure is plotted as a function of the radius (*D*/2) and the number of bandage layers. The thickness of each layer of the bandage ($t_{i}$) is 1 mm, and its width (*W*) is 10 cm. The results demonstrate that applied pressure can be controlled by adjusting the number of turns and the tension. But obviously, in real clinical settings, the direct implementation of this model clearly presents challenges for healthcare professionals. Therefore, a simpler method for measuring applied pressure is needed.

## 3. System Description

**Figure 2:**
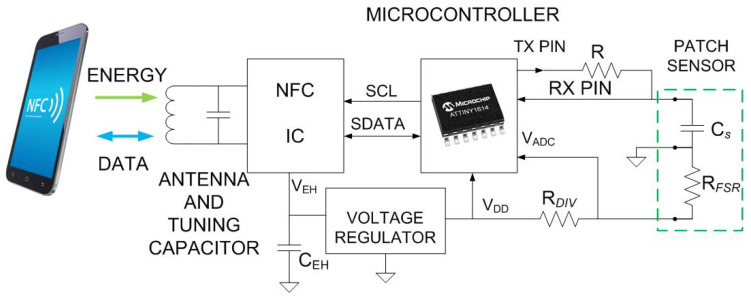
Block diagram of the system.

**Figure 3:**
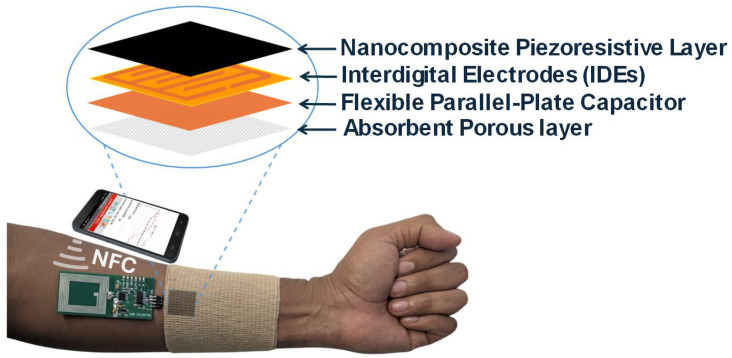
Schematic of the smart patch layers, their placement beneath the bandage, and their connection to the NFC board for smartphone readout.

Figure 2 shows the block diagram of the proposed wound monitoring system, which consists of two main parts: a smart patch formed of the pressure sensor and the capacitive sensor, and an NFC tag that reads the data from the sensors, stores it in its internal memory, and allows access to this information via a smartphone. The smart patch is connected to the NFC board through a header connector. Once measurements are completed, the NFC board can be disconnected to improve user comfort or can remain embedded beneath the bandage if necessary (Figure 3).

### 3.1. Smart Patch Design

#### 3.1.1. Pressure Sensor

**Figure 4:**
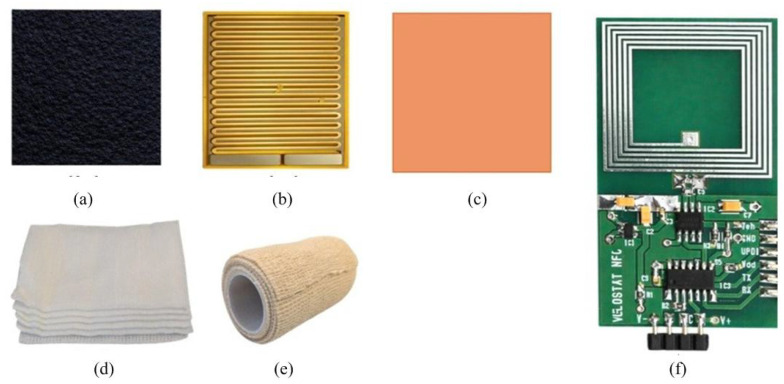
Physical components of the proposed smart bandage prototype: (**a**) pressure-sensitive resistive layer (Nanocomp), (**b**) interdigital electrodes (IDEs), (**c**) flexible parallel-plate capacitor with protective covers, (**d**) gauze for absorbent purposes, (**e**) compression bandage, and (**f**) NFC board.

A 20 mm square medical grade polymer containing carbon nanoparticles (Nanocomp) from Chip Quik Inc.,Ancaster, Canada, is used as the force-sensitive material (see Figure 4a). The polymer is a homogeneous conductor of electricity. Therefore, when it is compressed, its electrical conductivity changes. Using this material together with interdigital electrodes (IDEs), the resistance of the material, which decreases as pressure is applied to it can be measured (see Figure 4b). The interdigital electrode has a finger spacing of 0.254 mm and was fabricated on a flexible printed circuit board (PCB). The pressure-sensitive material was protected with a cellulose acetate cover. To this end a simple voltage divider is used. One terminal of the electrode is connected to ground, while the other is joined in series with a resistor whose other terminal is connected to $V_{\mathrm{DD}}$. A 100 kΩ resistor is selected for the voltage divider ($R_{DIV}$) to minimize current draw. If no pressure is applied to the material, it presents a very high impedance, on the order of megaohms. However, when compressed, its resistance decreases exponentially to the kiloohms range. The electrode voltage is read once it is connected to one of the analog inputs of the microcontroller that includes an internal ADC to measure that voltage ($V_{\mathrm{ADC}}$). The resistance of this force sensing resistor ($R_{FSR}$) is then calculated using the following expression:

(4) $$R_{FSR}=R_{DIV}\cdot \frac{V_{\mathrm{ADC}}}{V_{\mathrm{DD}}-V_{\mathrm{ADC}}}$$

The pressure values are obtained from ($R_{FSR}$) according to a previously obtained calibration table.

#### 3.1.2. Capacitance Moisture Sensor

**Figure 5:**
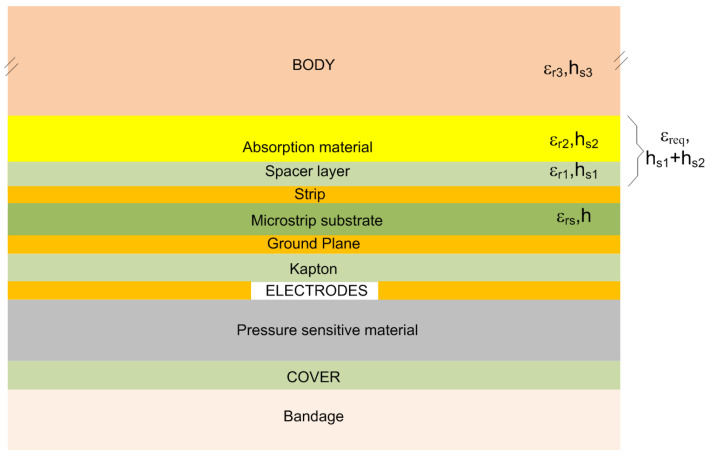
Cross-sectional diagram of the patch sensor.

A cross-section of the patch sensor is shown in Figure 5. The variations in the effective permittivity caused by wound exudate, that is absorbed in the absorption, modulate the capacitance between the body and the microstrip patch. This absorption layer also isolates the sensor from the body. The capacitance moisture sensor is based on a parallel-plate capacitor manufactured on a polyimide substrate, covered with a protective spacer layer made of cellulose acetate tape (see Figure 4c). This spacer layer (see Figure 4d) improves the biocompatibility of the patch and prevents short circuits between the conductive strips in the presence of conductive liquids (exudate). Moving towards the bandage (Figure 4e), the pressure-sensitive material is located between the electrodes printed on a flexible Kapton substrate and the bandage, resulting in a resistance that varies with the applied pressure. The patch was designed to be waterproof. To achieve this, the sensors are protected with a cellulose acetate coating. This protective layer ensures long-term stability and reliability. In principle, the patch could also be reused following appropriate disinfection.

For modeling purposes, the spacer and absorption layers are grouped together as a single stacked layer. An effective permittivity is defined for this stack, and the total thickness is taken as the sum of the thicknesses of the two layers.

The permittivity of the absorption layer changes as a function of the wound healing stage. The overall effective permittivity of the multilayer structure is approximated by modeling the layers as two series-connected capacitors:

(5) $$\varepsilon_{\mathrm{req}}=\frac{h_{s1}+h_{s2}}{h_{s1}\varepsilon_{r1}+h_{s2}\varepsilon_{r2}}$$

When the bandage is dry, the relative permittivity of the absorption layer is low (typically in the range 1.2–2 for textile or cellulose-based materials). However, when the layer becomes saturated with liquid due to exudation, its permittivity increases significantly. In this case, the effective permittivity is assumed to be approximated by the average of that of the liquid and that of the absorbent material, such that

(6) $$\varepsilon_{r2,\mathrm{wet}}≃\frac{1}{2}\left(\varepsilon_{r2,dry}+\varepsilon_{r,ex}\right)$$

where $\varepsilon_{r,ex}$ is the relative permittivity of the exudation liquid.

The capacitance can be estimated using the quasi-TEM approximation of a microstrip transmission line. The capacitance per unit length (F/m) of a microstrip line with a two-superstrate microstrip can be obtained from the effective permittivity:

(7) $$C^{\prime }\;=\;C_{\mathrm{air}}^{\prime }\;\varepsilon_{\mathrm{eff}}$$

The capacitance per unit length with all dielectrics replaced by air $C_{\mathrm{air}}^{\prime }$ is given by [37,38]:

(8) $$C_{\mathrm{air}}^{\prime }=\varepsilon_{0}\;\frac{W_{\mathrm{eff}}}{h}$$

where the effective width $W_{\mathrm{eff}}$ accounts for fringing electric fields by enlarging the physical strip width [37,38]:

(9) $$W_{\mathrm{eff}}=W+1.393h+0.667h\;\ln\;\left(\frac{W}{h}+1.444\right)$$

where *W* is the strip width, and *h* is substrate thickness.

The first term $\varepsilon_{0}W/h$ in (8) and (9) corresponds to the capacitance of a parallel-plate structure, while the second term accounts for the additional capacitance due to fringing fields.

The filling factors provide useful insight into the distribution of energy in an inhomogeneous transmission line and, therefore, about the contribution of each layer. The effective relative permittivity can be expressed in terms of the filling factors $q_{i}$ [37,39,40]:

(10) $$\varepsilon_{\mathrm{eff}}=q_{1}\;\varepsilon_{rs}+q_{2}\;\varepsilon_{req}+q_{3}\;\varepsilon_{r3}$$

where $\varepsilon_{ri}$ are the relative permittivities of each layer. The filling factors for the substrate ($q_{1}$), the equivalent layer composed by the spacer material (superstrate layer 1) and the absorption layer (superstrate layer 2) ($q_{2}$), and the body (superstrate layer 3) ($q_{3}$) are given by [37,39,40]:

(11) $$q_{1}=\frac{1}{2}\left(1+\frac{1}{\sqrt{1+12\frac{h}{W}}}\right)$$

(12) $$q_{2}=\frac{1}{2}\left(1-\frac{1}{\sqrt{1+12\frac{h}{W}}}\right)\left(1-e^{-(h_{s1}+h_{s2})/h}\right)$$

(13) $$q_{3}=1-q_{1}-q_{2}$$

The variation in capacitance as a function of the moisture content of the absorption layer is given by:

(14) $$\Delta C=L\left(C_{wet}^{\prime }\cdot x-C_{dry}^{\prime }\cdot \left(1-x\right)\right)$$

where *L* is the length of the patch, and $C_{dry}^{\prime }$ and $C_{wet}^{\prime }$ are the capacitances per unit length calculated from the dry absorption material or saturated with exudation liquid (wet state) given by (5). The fraction of area occupied by the exudate material is denoted by *x* (0≤x≤1).

The measurement of capacitance is performed in time-domain based on the step response, therefore the bandwidth of the system is about 0.35 times the inverse of the rise time, which is on the order of the RC time constant. Therefore, permittivity data should be considered in the kHz–MHz frequency range. In this range, the permittivity of biological tissues is very high due to ionic and interfacial polarization mechanisms. The relative permittivity of the exudate material, which is very high and on the order of plasma or water, depending on its composition.

As the patch must be flexible, the substrate must be thin. The use of a thin substrate has another advantage, since its thickness is much less than the width of the patch (h<<W). Therefore, the filling factor $q_{1}$ is close to one. The filling factor $q_{2}$ depends on the thickness of the absorption layer $h_{s1}$. However, when the thickness of superstrate is approximately four times the thickness of the substrate, the filling factor $q_{2}$ becomes almost constant. In this case, the filling factor associated with the body layer, $q_{3}$, tends to zero, resulting in reduced sensitivity to the body’s permittivity especially in the case of thick absorbing layers.

Simulations are performed to estimate the maximum change in capacitance between the dry and wet states. Table 2 summarizes the dielectric properties and thicknesses of the microstrip layers. The composition of wound exudate varies depending on the stage of healing and the condition of the tissue. Wound exudate is a complex biological fluid [41], but it is generally composed of water and electrolytes, which provide the conductivity of the medium, proteins, cellular components and biomolecules. In this simulation, the liquid is treated as plasma, since blood is the main component of the exudate. Consequently, the simulated conductivity is 1.0 S/m, which is consistent with that of blood, which in the low-frequency range lies between 0.25–1 S/m [42]. The permittivity of the absorption layer saturated with the exudate material is assumed to be the average of the permittivities of the exudate material and the absorption material, the relative permittivity of the latter being approximately 2 and its conductivity low. Wound exudate increases the effective permittivity of the sensing region due to the high dielectric constant of biological fluids [43]. In the electromagnetic simulations, the conductivity is taken as 0.5 S/m. The thickness of the body layer is approximated by the fringing-field penetration height into the infinite superstrate, approximated by *W*/2. At low frequencies, the relative permittivity of skin and fat is generally lower than that of muscle [44]; however, in the simulations these values are assumed to be equal to 100. On the other hand, due to the low filling factor $q_{3}$, the contribution of the body is small for the dry case and considerably less than the wet absorption layer, which has a high permittivity and is closer to the electrodes.

**Figure 6:**
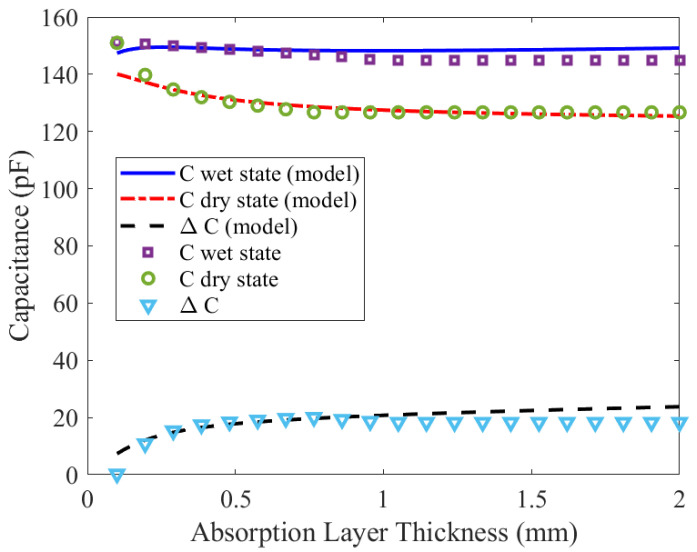
Comparison between the capacitance model and full-wave simulations. Capacitance in dry and wet conditions, and their difference, as a function of the absorbent layer thickness.

Figure 6 shows both the capacitances for the dry and wet states and the difference between them as a function of the thickness of the absorption layer. These capacitances are calculated using (14). The results obtained with the model have been compared with electromagnetic simulations performed with PathWave Momentum (3D Planar EM Simulator) from Keysight Technologies, Inc.Santa Rosa, CA, USA. Good agreement has been obtained confirming the independence of the absorption layer thickness when determining capacitance. With the considered parameters, the capacitance variation between the dry and wet (saturated) states is approximately 15%. Capacitance for the dry state remains almost unaffected by variations in the body’s permittivity and can therefore be used as a reference for normalizing the capacitance measurements.

### 3.2. NFC Tag

The proposed NFC module, shown in Figure 4f consists, of an NFC IC with energy-harvesting capability (ST25DV04K, STMicroelectronics, Plan-les-Ouates, Switzerland), an NFC antenna, a microcontroller (ATTiny1614, Microchip Technology, Chandler, AZ, USA), a regulator (TSP7627, Texas Instruments, Dallas, TX, USA), a storage capacitor and a charge resistance. The physical components employed in the fabrication and experimental validation of the proposed smart bandage prototype are shown in Figure 4, including the multilayer sensing structure and the NFC electronic module (Figure 4f). The multi-layer sensing structure interacts with the NFC unit, enabling simultaneous monitoring of pressure changes. The ST25DV04K complies with the ISO/IEC 15693 standard and NFC Forum Type 5 specifications, which define modulation parameters, and supports energy harvesting. Previous work has demonstrated that this device can harvest more than 3 V and 3 mA from a printed square loop with dimensions up to 3 cm on each side [45].

#### 3.2.1. NFC Antenna Tuning

In this work, an antenna with a side length of 2.5 cm and six turns is selected, resulting in an inductance of 1.16 μH. The trace width and inter-trace spacing were both set to 0.5 mm. The prototype is fabricated on an FR4 PCB with a thickness of 0.8 mm. Therefore, the structure is semi-rigid, which helps prevent mechanical damage to the components and avoids issues associated with bending [46,47,48]. To maximize energy transfer, the resonant frequency of the tag must be tuned to the NFC operating frequency of 13.56 MHz. Accordingly, a tuning capacitor of 69 pF is connected in parallel with the antenna loop at the input of the NFC IC. The resonant frequency of the tag can be estimated using [22]:

(15) $$f_{r}≃\frac{1}{2\pi \sqrt{L_{a}\left(C_{IC}+C_{p}+C_{tuning}\right)}}$$

where the chip capacitance $C_{IC}$ is nonlinear but remains approximately constant for typical input power ranges (28.5 pF for the ST25DV04). The parasitic capacitance $C_{p}$ consists of the antenna’s parasitic elements, including interconnections, and is on the order of a few picofarads. Fine tuning can be performed on the prototype by measuring the resonance frequency using a vector network analyzer (VNA) connected to a test loop. A peak in the measured $S_{11}$ parameter indicates the resonance frequency, provided that the test loop is placed at an appropriate distance to avoid excessive coupling. A ground plane located at a representative reading distance from the antenna can be used to emulate the presence of a mobile device during tuning process.

**Figure 7:**
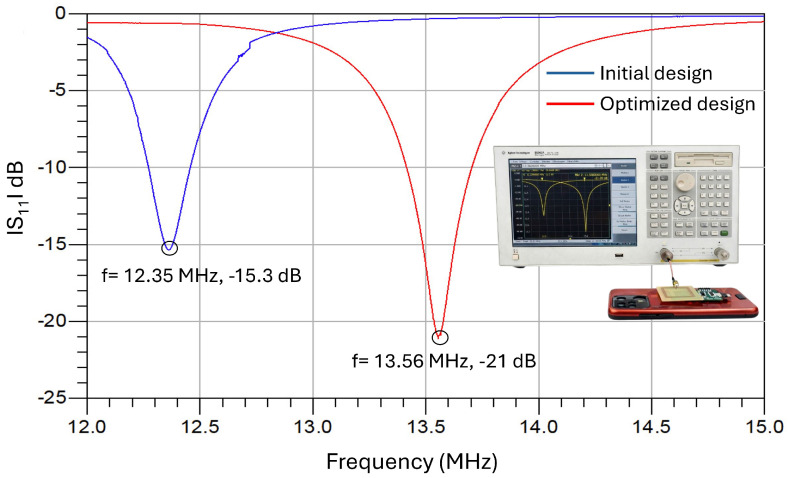
Measured reflection coefficient $S_{11}$ of the NFC antenna for the initial (in air) and optimized designs under representative reader-load conditions (in close proximity to a smartphone).

Figure 7 shows the measured reflection coefficient $S_{11}$ of the NFC antenna for the initial and optimized designs under representative reader-load conditions. The resonant frequency shifts from 12.35 MHz to 13.56 MHz because of magnetic coupling and loading. This confirms that the antenna is properly tuned to the NFC standard frequency under realistic operating conditions, ensuring efficient impedance matching and power transfer.

This approach accounts for detuning effects caused by the metallic parts in the layout as well as by the proximity of the mobile device [22]. In addition, this procedure eliminates the need for a high-precision antenna model. Only the approximate value of the antenna inductance needs to be known, which can be obtained using empirical formulas such as Wheeler’s [49]. Since the body does not exhibit magnetic properties, the proximity of the tag does not affect the antenna’s inductance; it only changes the parasitic capacitance $C_{p}$. This effect is irrelevant because $C_{p}$ is much smaller than the IC and tuning capacitances. Biological tissues exhibit high permittivity and conductivity, which significantly affect antennas operating in the UHF and GHz bands through dielectric loading and increased electromagnetic losses [50,51,52]. Conversely, NFC systems operating at 13.56 MHz rely predominantly on magnetic near-field coupling, which is intrinsically less sensitive to tissue permittivity and maintains stable performance in wearable scenarios, including those subjected to mechanical deformation [53]. Therefore, NFC technology provides a more robust solution for on-body and bandage applications compared to UHF RFID [54] and higher-frequency wireless systems such as Bluetooth [55].

The system has been designed to allow reuse of the NFC board and, therefore, only the bandage is disposable. However, if the NFC board were to be integrated directly within the bandage, user comfort could be improved by fabricating the electronics on a flexible substrate, such as polyimide or Polyethylene Terephthalate (PET).

Bending of the antenna can reduce the magnetic coupling and detune the tag due to variations in the antenna inductance [46]. In contrast, the effect of bending on the parasitic capacitance is relatively small, since the antenna parasitic capacitance is typically only a few picofarads, significantly lower than the tuning capacitance and the IC input capacitance. These detuning effects can be compensated by adjusting the tuning capacitance using the vector network analyzer (VNA) procedure described previously. For short-range operation, the variation in coupling caused by bending is considered a second-order effect compared with the reduction in antenna inductance produced by the proximity of metallic parts of the mobile device. Bending may reduce the read range by approximately 5 mm; however, this effect remains limited when the tag is operated by tapping the mobile device at short distance. The use of conventional metallic conductors (e.g., aluminum or copper), compatible with standard flexible PCB manufacturing techniques, is preferable to ink-based printed conductors. This approach avoids several reliability issues associated with printed conductive inks, such as trace cracking under bending and difficulties in soldering electronic components [47,48].

**Figure 8:**
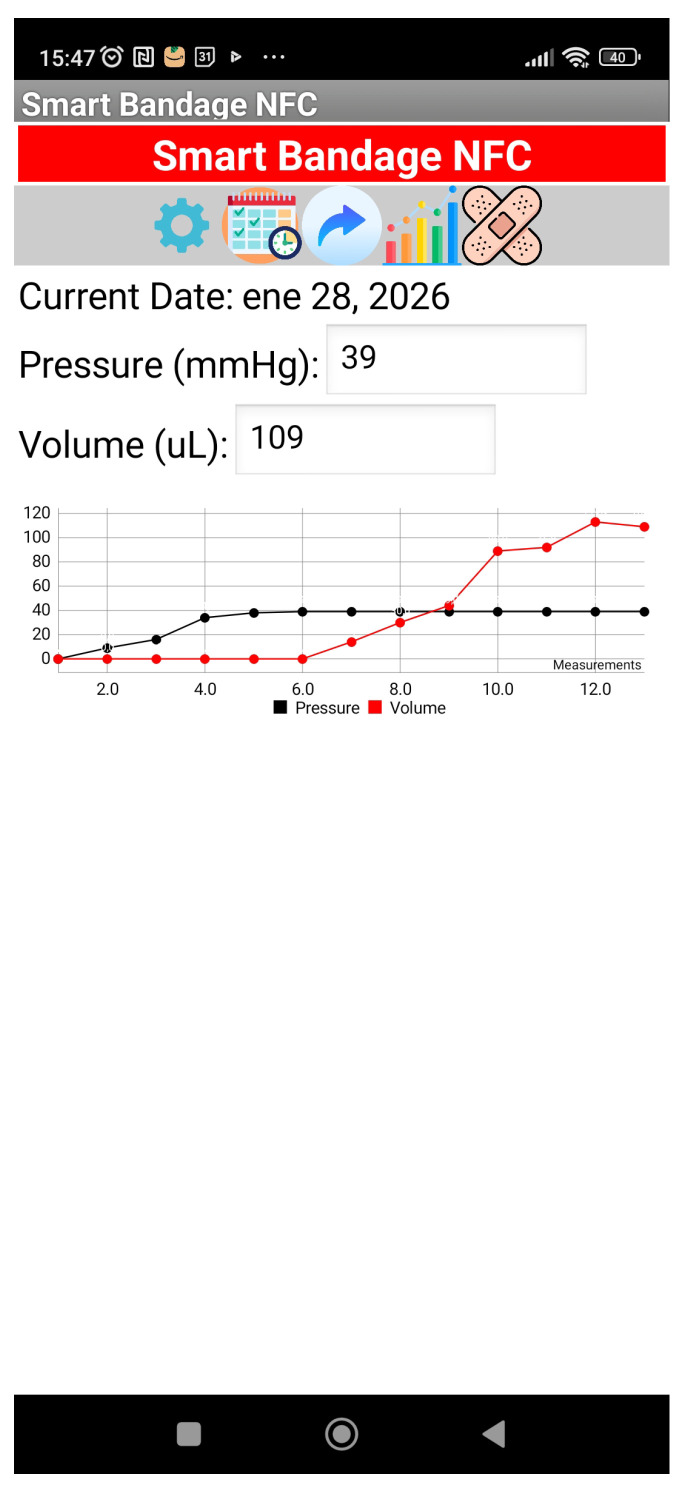
Screenshot of the Android-based demonstration application developed to test the system.

An Android-based application has been developed to test the system (see screenshot in Figure 8). The successive measurements are displayed on the screen, showing both the measured pressure and the estimated volume of exudate. The data are stored in a Google sheet, along with information about the user and the date of the measurements.

#### 3.2.2. Capacitance Sensor Measurements

To monitor the condition of the wound, the capacitive sensor employs two parallel electrodes that detect changes caused by exudate levels, which makes it possible to determine the wound’s healing state based on a predefined capacitance threshold.

**Figure 9:**
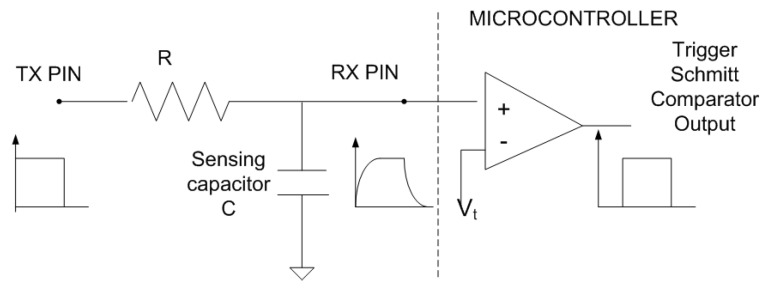
Scheme of the circuit used for measuring the capacitance.

**Figure 10:**
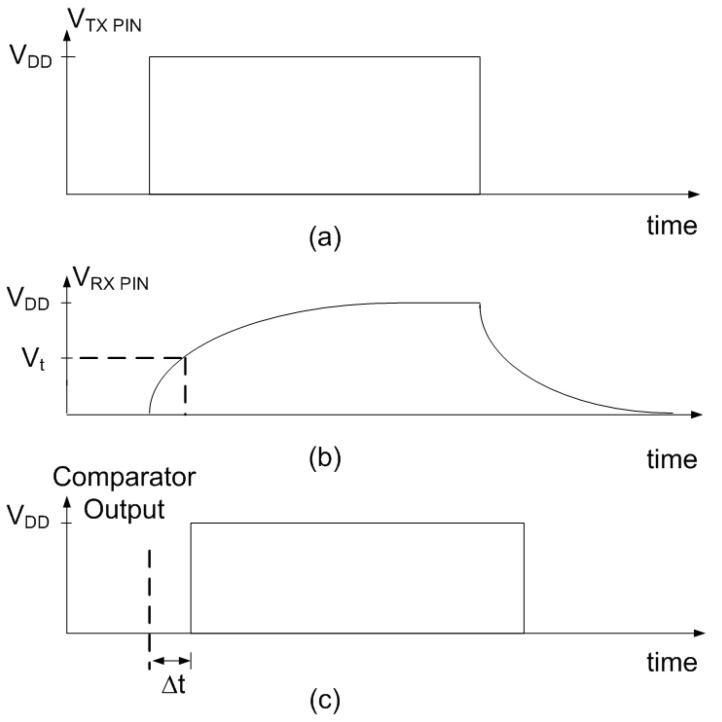
(**a**) Pulsed excitation applied to the TX pin. (**b**) Voltage at the RX pin in response to the applied step. (**c**) Output of the microcontroller’s internal comparator. The delay Δt is proportional to the RC charge time constant.

Commercial capacitance-to-digital converters (CDCs), such as the AD7746 (Analog Devices, Wilmington, MA, USA) and the FDC1004 (Texas Instruments, Dallas, TX, USA), provide high accuracy, on the order of femtofarads. However, their integration into the proposed battery-less NFC system is challenging due to supply voltage requirements (3.3 V in the case of the FDC1004) or limited measurable capacitance range (4 pF in the case of the AD7746). To overcome these limitations, capacitance is measured by evaluating the RC time constant using the microcontroller [56]. This approach also reduces component count and lowers the overall cost of the prototype. This measurement method only requires the General Input/Output (GPIO) pins of the microcontroller and an external resistance (*R*). Therefore, it does not add any active components that could increase power consumption. The charging time of the capacitive electrode is measured using a high-value resistor *R* (10 MΩ in the prototype) connected to one of the microcontroller’s digital outputs (TX pin) (see Figure 9). The microcontroller firmware sets the TX pin from low to high, monitors a digital input (RX pin), and waits until its logic level matches that of the TX pin (see Figure 10). An integer counter is incremented within a while loop until the state of the RX pin changes. The resulting count value is therefore proportional to the delay between the two pin transitions and, consequently, to the capacitance of the sensor.

The delay Δt between the state changes of the TX and RX pins is determined by the RC time constant, where C is the total capacitance at the receive pin, including the electrode and parasitic capacitances. The delay (rise time) can be computed as:

(16) $$\Delta t=-RC\cdot \ln\left(1-\frac{V_{t}}{V_{DD}}\right)$$

ST25DV04K dynamic NFC/RFID tag IC with 4-Kbit EEPROM (STMicroelectronics, Marseille, France).

In this expression, $V_{t}$ denotes the threshold voltage of the microcontroller’s digital input when the RX pin is configured as a digital input. It corresponds to the input high voltage, specified as $0.7V_{DD}$ in the microcontroller datasheet. $V_{DD}$ is the digital supply voltage provided by the regulated energy-harvesting output of the NFC IC. Since the energy-harvesting output of the NFC IC used (ST25DV04K from STMicroelectronics, Marseille, France) is unregulated, a TPS76927 low-dropout 2.7 V linear regulator (Texas Instruments Inc., Dallas, TX, USA) is employed to stabilize the supply, together with a 1 μF storage capacitor ($C_{EH}$), which supports a voltage drop of 20 mV at the regulator input. This value is acceptable for low-dropout regulators as long as the load currents are less than 2 mA [57]. The measurement resolution is determined by the minimum time step detectable by the ATtiny1614 microcontroller (Microchip Technology Inc., Chandler, AZ, USA), which is limited by the system clock frequency and internal processing delays. To reduce power consumption, the prototype operates at a clock frequency of 4 MHz. Although the proposed method is simple, it provides sufficient precision for the intended application. The accuracy of the capacitance measurement can be further improved by increasing the value of the resistor in Equation (16). Note that, since the threshold voltage is related to $V_{DD}$, the term $\log\;\left(1-V_{t}/V_{DD}\right)$ in Equation (16) is independent of the microcontroller bias conditions. In addition, this factor does not need to be explicitly known, as the normalized capacitance values are sufficient to estimate the liquid volume. For production volumes exceeding 100 units, the total Bill of Materials (BOM) and fabrication cost for the NFC sensing board is estimated at under 3 € per unit.

**Figure 11:**
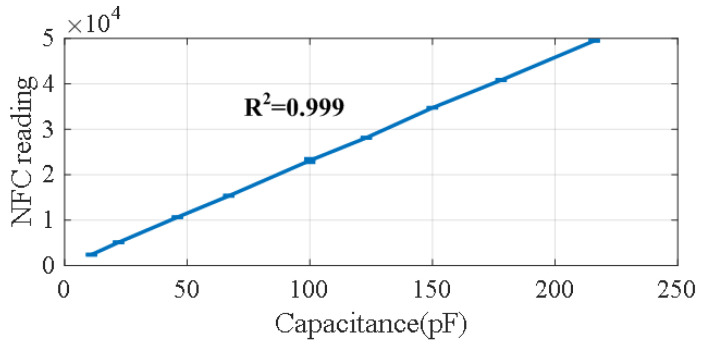
Measured counter output as a function of capacitance. Error bars indicate the standard deviation computed from 10 repeated measurements.

To demonstrate the accuracy of the proposed method for measuring capacitance, discrete capacitors with a tolerance of 5% are measured using the NFC device. Figure 11 shows the average NFC reading (the output of the counter measuring the delay) as a function of capacitance. A linear regression is applied, yielding a correlation coefficient close to one. The slope of the regression is used to convert the NFC readings to capacitance values, while the parasitic capacitance introduced by the interconnection wires is obtained from the intercept. The maximum deviation between the measured and nominal capacitance is 1.47%, which falls within the specified capacitor tolerance.

## 4. Results and Discussion

### 4.1. Pressure Sensor Characterization

In polymers filled with carbon nanoparticles (such as carbon black, carbon nanotubes, or graphene), the electrical conductivity increases under pressure due to reduced distance between particles, improved formation of the conductive network, and electron tunneling effects [58,59,60].

**Figure 12:**
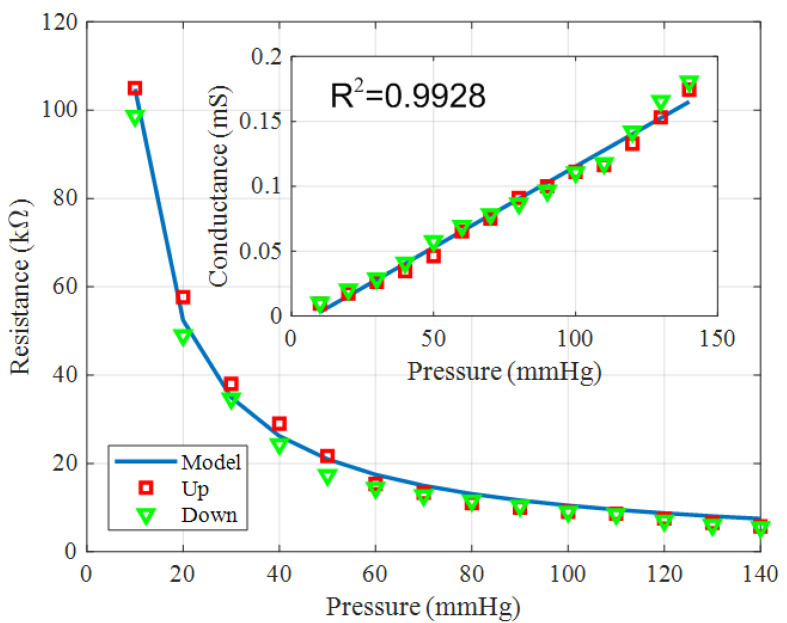
Measurements of the resistance of the force-sensitive resistor as a function of the applied pressure (mmHg), together with the fitted model. The inset shows the conductance as a function of pressure along with the corresponding fitted model.

Figure 12 shows the measured resistance of a force-sensitive resistor with an active area of 2cm×2cm as a function of the applied pressure. The inset displays the corresponding conductance, defined as the inverse of the resistance.

Based on these results, the resistance of the pressure sensor is empirically modeled as the sum of a parasitic resistance and a term inversely proportional to the applied pressure *P* that considers takes into account the material’s conductance as a function of the applied pressure [58]:

(17) $$R_{FSR}=R_{s}+A/P$$

where $R_{s}$ denotes the parasitic resistance and *A* is a fitting parameter. Both parameters are obtained via linear regression with respect to the inverse pressure, $P^{-1}$. The extracted slope is $A=1.048\times 10^{6}\;\Omega \cdot \mathrm{mmHg}$, and $R_{s}$ is close to zero.

Figure 12 compares the average of three measurements with the modeled response of a force-sensitive resistor with an active area of 2cm×2cm. The inset in Figure 12 confirms that the conductance increases with applied pressure. The correlation coefficient between the model and the measured conductance was $R^{2}=0.99$.

**Figure 13:**
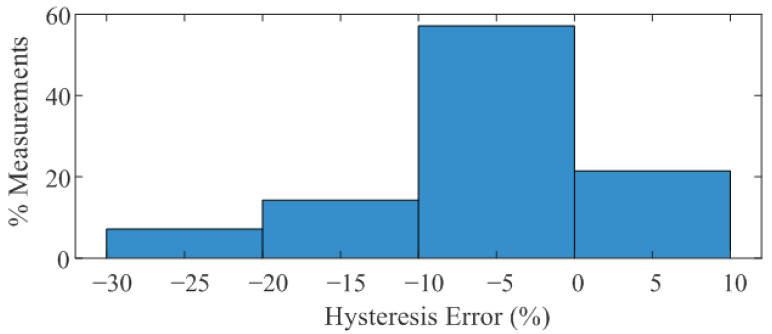
Histogram of hysteresis error, defined as the normalized difference in conductance between upward and downward sweeps.

To account for bending effects, measurements were performed with the pressure sensor positioned around a volunteer’s arm. Slight hysteresis was observed during loading and unloading. Figure 13 shows a histogram of the hysteresis, defined as the normalized variation in conductance between upward and downward sweeps. The error is typically within 5–10%, and the observed variability is mainly attributed to uncertainties in the pressure measurements provided by the sphygmomanometer, associated with slight pressure losses after the target pressure is established. To improve calibration accuracy, commercial pneumatic devices such as the Kikuhime system developed by MediGroup/TT Meditrade (Birkerød, Denmark) [61,62] or the PicoPress system developed by Microlab (Padova, Italy) [63] could be used in future work and clinical studies.

For the pressure sensor, the sensitivity—defined as the slope of the calibration curve (Figure 12) obtained through linear regression—was 1.25 mS/mmHg. The limit of detection (LOD) was estimated using the 3σ/S criterion, where σ represents the standard deviation of the samples (0.45 mS) and *S* is the sensitivity, yielding an approximate LOD of 1 mmHg.

**Figure 14:**
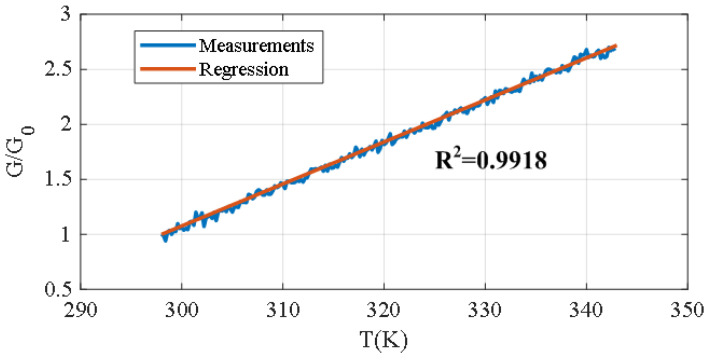
Normalized conductance with respect to the reference temperature as a function of temperature, together with the regression fit.

The influence of temperature has been investigated by attaching a force-sensitive resistor (FSR) to a hot plate and characterizing its resistance as a function of temperature using a multimeter connected to a computer. A temperature sensor located close to the FSR (PT100) was used to measure the temperature with a second multimeter. A weight was placed on the sensor to apply a pressure of approximately 40 mmHg. Figure 14 shows the normalized conductance as a function of temperature. A linear dependence was obtained, in agreement with the theoretical results reported in the literature [64]. A linear model was proposed to describe the observed behavior:

(18) $$G=1/R_{FSR}=G_{0}\left(1+\alpha \left(T-T_{0}\right)\right)$$

where *G* is the conductance and $G_{0}$ is the conductance at the reference temperature $T_{0}=298\;K$. The thermal coefficient α obtained from the fit is $0.038\;K^{-1}$.

Based on the pressure sensitivity, a temperature variation of 1 ℃ results in an estimated pressure variation of approximately 1.52mmHg for pressures around 40mmHg. This variation is acceptable, considering that the temperature under the bandage remains nearly constant and close to body temperature.

**Figure 15:**
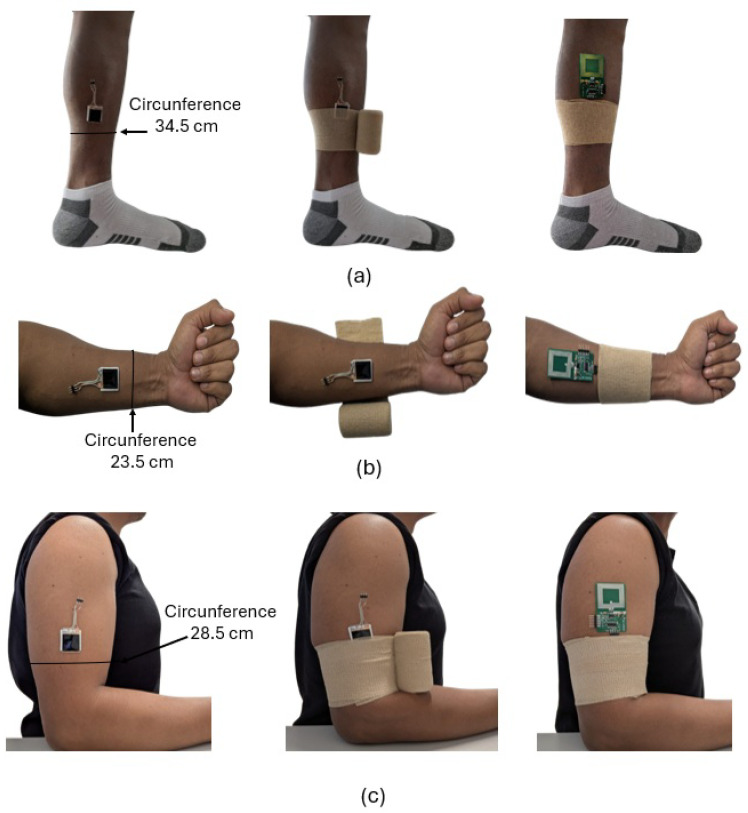
Smart bandage placement on (**a**) lower leg, (**b**) wrist, and (**c**) upper arm (biceps). The perimeters measured in each case are indicated.

**Figure 16:**
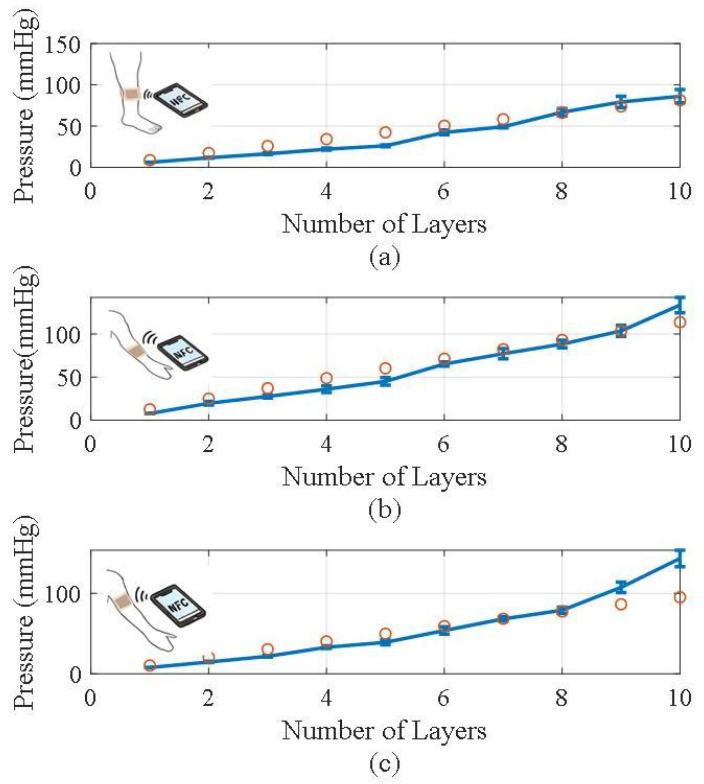
Measured pressure as a function of the number of bandage layers: (**a**) lower leg, (**b**) wrist, and (**c**) upper arm (biceps). The error bars denote the standard deviation of the measurements. The circular symbols represent the values obtained from the thick wall cylinder theory assuming a tension of 6.5 N.

Experiments to evaluate the performance of the pressure sensor on different parts of the upper limbs (wrist and biceps) and lower limbs (soleus) are conducted. According to the theoretical model, the pressure exerted by the bandage depends mainly on the local diameter of the limb and the tension applied during compression. Figure 15 shows the experimental setup used for each sensor location: (a) soleus, (b) wrist, and (c) biceps, consisting of the following components: sensors, a compression bandage, and an NFC module. The perimeter of the measurement region is also shown. To experimentally validate this relationship, the number of layers of the bandage in each anatomical location is increased in a controlled manner. Figure 16 shows the average pressure exerted by the bandage as a function of the number of layers applied, along with the corresponding standard deviation, obtained over a 10 min interval using the proposed prototype and a smartphone (model Xiaomi Redmi 9) as the NFC reader. A comparison with the theoretical model given by Equation (1) has been included. It is considered a constant tension of $T_{i}$ = 6.5 N. As expected, the measured pressure increases with the number of layers of bandage, in accordance with the predictions of the theoretical compression model [35] described in Section 2 The discrepancies observed, particularly when the number of turns is higher, are mainly attributed to the difficulty of maintaining the same tension for each turn during the winding process.

### 4.2. Capacitive Sensor Characterization

To emulate wound exudate, a 0.1 M phosphate-buffered saline (PBS) solution is used. To increase the electrical conductivity, 0.9wt% NaCl is added, resulting in a conductivity of 1.5S/m. The solution is introduced beneath the absorption layer using a pipette.

**Figure 17:**
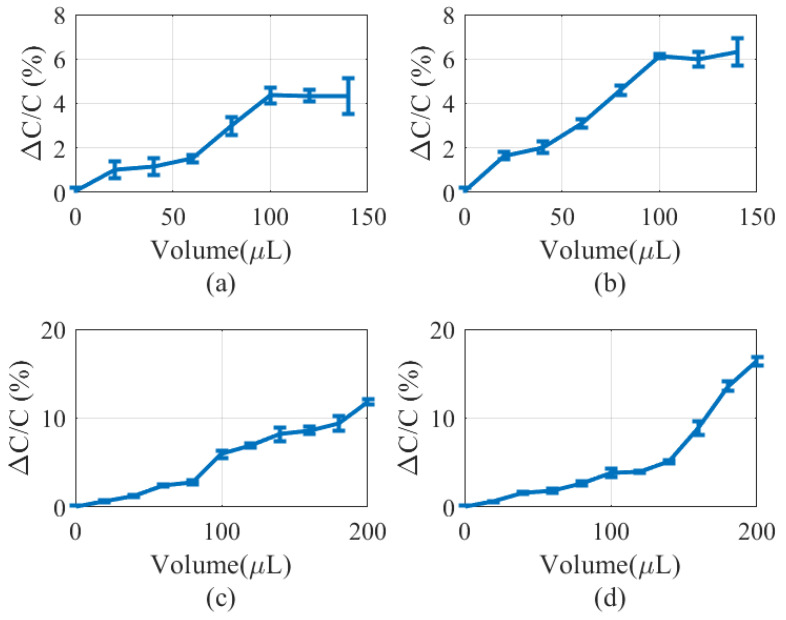
Capacitance variation as a function of the absorbent layer under an applied pressure of 40 mmHg: (**a**) 1 mm thick absorbent paper, (**b**) 1.5 mm thick absorbent paper, (**c**) 1 mm-thick gauze, and (**d**) 2 mm thick gauze.

Figure 17 shows the capacitance variation for different materials and thicknesses of the absorbent layer. The capacitance is obtained from the NFC readout one minute after the application of the liquid to ensure complete diffusion within the absorbent layer. Ten measurements are taken to determine the deviation represented by the error bars. The approximate liquid volumes before saturation of the samples, when using cellulose (absorbent paper) or cotton gauze, were approximately 100μL and 150μL, respectively.

When fluid wound exudate is absorbed into a layer of paper or gauze, the process is mainly governed by diffusion and capillary absorption within the material’s porous structure. These materials are composed of cellulose or cotton fibers containing numerous microscopic pores. When the liquid comes into contact with the surface, it penetrates the pores through capillary forces. Subsequently, the liquid molecules diffuse from regions of higher concentration to those of lower concentration. Therefore, the area and thickness of the wet region of the absorption layer expand over time until equilibrium is reached.

**Figure 18:**
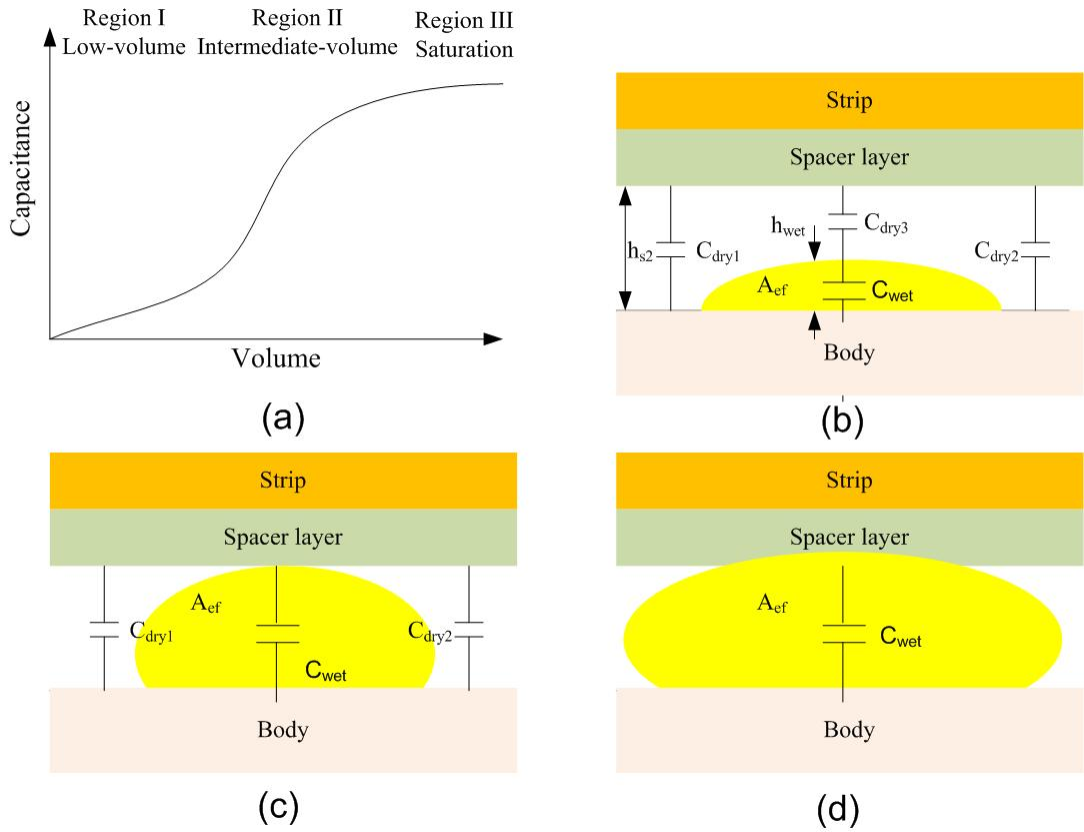
(**a**) Schematic illustration of capacitance as a function of volume, showing the three characteristic regions, together with a cross-sectional view of diffusion within the absorption layer: (**b**) Region I: low-volume regime, (**c**) Region II: intermediate-volume regime, (**d**) Region III: saturation regime.

The variation in the system’s capacitance is modeled electrically as a combination of three capacitors connected in parallel, two of which are associated with the dry regions $C_{dry1}$ and $C_{dry2}$, and a third that has an equivalent capacitance formed by the series combination of $C_{dry3}$ and $C_{wet}$, corresponding to the dry and wet regions, respectively (see Figure 18). Since the permittivity of the wet region is significantly higher than that of the dry region, the overall capacitance increases as the wetted area expands.

The effective capacitance of the absorption layer $C_{abs}$ is given by:

(19) $$C_{abs}=\frac{\epsilon_{0}\epsilon_{req,abs}A}{h_{s2}}=C_{dry1}+C_{dry2}+\frac{1}{1/C_{dry3}+1/C_{wet}}$$

where *A* is the patch area, $\epsilon_{req,abs}$ is the equivalent relative permittivity of the absorption layer. The capacitance of the dry region of the absorption layer can be expressed as:

(20) $$C_{dry1}+C_{dry2}=\frac{\epsilon_{0}\epsilon_{r2}\left(A-A_{ef}\right)}{h_{s2}}$$

(21) $$C_{dry3}=\frac{\epsilon_{0}\epsilon_{r2}A_{ef}}{h_{s2}-h_{wet}}$$

The capacitance of the region containing wound exudate is given by:

(22) $$C_{wet}=\frac{\epsilon_{0}\epsilon_{r,ex}A_{ef}}{h_{wet}}$$

In the capacitance measurements as a function of volume, three distinct regions can be identified. In the first of these (region I), the volume of fluid is relatively small, remains concentrated around the wound, and does not reach the electrodes. As the volume increases under this condition, the height of the dry region below the wound ($h_{s2}-h_{wet}$) decreases while the effective spread area $A_{eff}$ increases. This results in an approximately linear increase in capacitance.

The second region (region II) begins when the liquid reaches the electrodes ($h_{wet}=h_{s2}$). In this regime, the increase in capacitance is primarily driven by the expansion of the wetted area $A_{eff}$, leading to a change in the slope of the capacitance–volume curve.

The final region corresponds to the saturation of the absorption layer (region III). In this regime, the liquid progressively covers the entire absorption layer and the capacitance tends to saturate and remain approximately constant. The slopes in the different regimes depend on the diffusion coefficient of the material as well as its absorption capacity and porosity.

The thickness of the absorbent layer strongly affects the diffusion process. In thinner layers, absorption occurs more rapidly because the liquid travels a shorter distance. As a result, saturation is reached earlier, although the total saturation volume is lower than in thicker layers. This behavior can be observed in Figure 17a and Figure 17b for absorbent papers 1 mm and 1.5 mm thick, respectively. Similarly, the saturation volume is higher in the thick gauze (Figure 17d) than in thin gauze (Figure 17c), while saturation occurs earlier in the thinner gauze samples.

For both materials, an approximately linear response is observed at low liquid volumes, which is associated with dominant vertical diffusion from the bottom toward the upper layers of the material. Once the liquid reaches the electrode region, a rapid increase in capacitance is observed in all cases. Subsequently, the increase in capacitance is governed primarily by lateral diffusion within the absorbent layer until saturation is reached.

Due to the complexity involved in developing a complete physical model, a compact empirical expression that reasonably fits the measured capacitance as a function of volume is used in this work to estimate the volume of wound exudate in the absorption layer.

**Figure 19:**
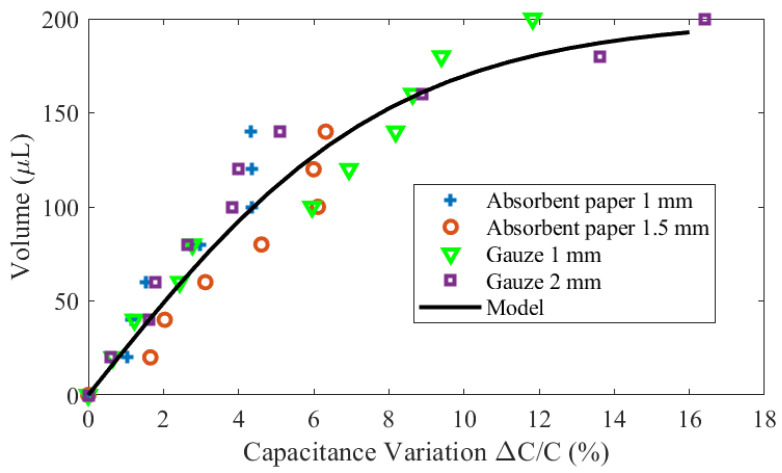
Volume estimated from the measured capacitance variation under an applied pressure of 40 mmHg.

Figure 19 shows the estimated volume as a function of the measured capacitance for different materials and absorption layer thicknesses. A simple model is proposed using an interpolation function based on a hyperbolic tangent:

(23) $$V=V_{sat}\cdot \tanh\left(\frac{\Delta C/C}{\alpha \Delta C_{0}/C_{0}}\right)$$

where $V_{\mathrm{sat}}$ is the saturation volume, ΔC/C is the normalized capacitance variation (in %) at the end of the linear range, and α is a parameter that controls the saturation rate. For $\Delta C_{0}/C_{0}<\Delta C/C$, the hyperbolic tangent can be approximated by a linear function, allowing $\alpha \;\Delta C_{0}/C_{0}$ to be extracted from the inverse slope of the linear region by linear regression. These parameters are obtained by fitting the experimental data, yielding $V_{\mathrm{sat}}=200\;\mu L$, $\Delta C_{0}/C_{0}=4\%$, and α=2. Good agreement is obtained using the proposed model. The standard deviation of the error is less than 15μL for all absorbent materials. The maximum variation between the saturated (wet) and dry states is in the order of 15%, consistent with the values predicted by simulations.

Within the linear operating range, which corresponds to a relative capacitance variation less than 8%, the sensor has a sensitivity of 19.3μL/(%ΔC/C). The limit of detection (LOD), estimated from the standard deviation of the measurements (17.3μL), is 2.6μL.

**Figure 20:**
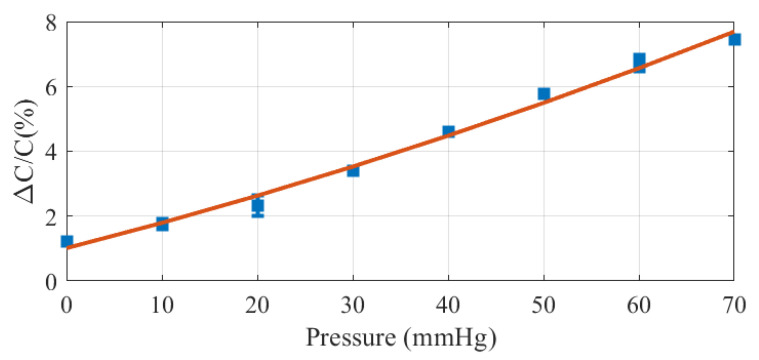
Normalized capacitance variation as a function pressure for a volume of 100 μL.

The influence of the applied pressure on the capacitance measurements is investigated in the following figures. Figure 20 shows the capacitance variation as a function of pressure for a fixed liquid volume of 100μL. Since the absorbent layer is flexible and compressible, increasing in pressure reduces its thickness and increases the wetted area. Consequently, the capacitance increases with pressure.

**Figure 21:**
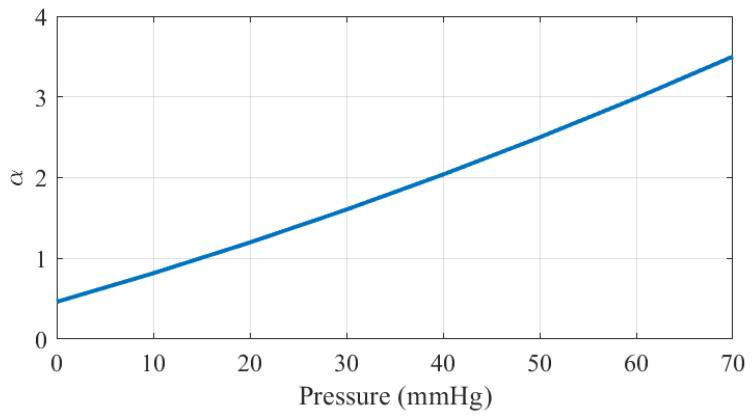
Parameter α as a function of the pressure in mmHg.

To incorporate this phenomenon into the proposed empirical model, it is assumed that the saturation volume does not depend on pressure. Under this assumption, the fitting parameter α, which governs the slope of the curve in the linear regime, is extracted from the data shown in Figure 20. As illustrated in Figure 21, α exhibits an approximately linear dependence on pressure.

**Figure 22:**
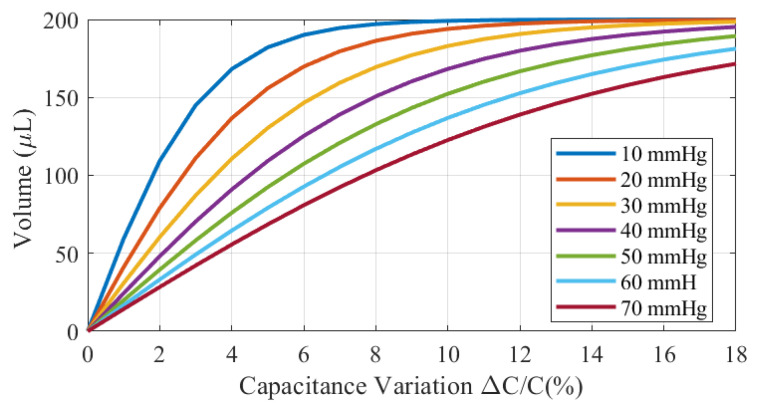
Volume estimated from measured capacitance variation for different pressures.

By considering the parameter α as a function of pressure, the absorbed liquid volume can be estimated from both the measured capacitance variation and the applied pressure (see Figure 22). The model can then be used to define a threshold capacitance variation for a given pressure. Beyond this threshold, the bandage is considered to need to be replaced due to the presence of wound exudate.

**Figure 23:**
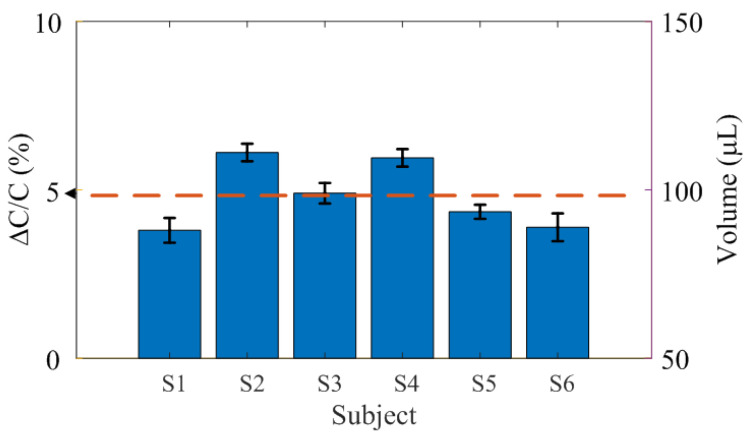
Measured capacitance variation for each patient. Error bars represent the standard deviation of five measurements. The mean value is indicated by the dashed line.

To study the variation in measurements among patients, measurements are performed on six volunteers after applying 100μL of saline solution at a nominal pressure of 40mmHg. A cotton gauze pad approximately 2mm thick is used. Measurements are repeated five times for each patient. Figure 23 shows the normalized variation of the measured capacitance and volume, where the error bars denote the standard deviation. The average deviation between patients is 17.5 μL. The average value agrees with the model-based estimate.

The volume of sweat produced per unit area of skin, commonly referred to as the local sweat rate, depends primarily on factors such as temperature, physical activity, and body region [65,66]. Typical local sweat rates on the arm range from 0.1–0.5 mg·${\mathrm{cm}}^{-2}\cdot$$\min^{-1}$ at rest, 0.5–2.0 mg·${\mathrm{cm}}^{-2}\cdot$$\min^{-1}$ during moderate exercise or mild heat exposure, and 2–5 mg·${\mathrm{cm}}^{-2}\cdot$$\min^{-1}$ during intense exercise or hot environmental conditions [15]. For a patch area of 4 cm ^2^, the accumulated sweat volume over a 10 min period is estimated to be 4–20 μL at rest, 20–80 μL during moderate exercise, and up to 80–200 μL during intense exercise. Under resting conditions, the sweat volume remains below the minimum detectable threshold determined in previous experiments. Nevertheless, moderate sweating can still be reliably detected. Since users can readily recognize these conditions, periodic replacement of the bandage is recommended to reduce the risk of infection.

**Figure 24:**
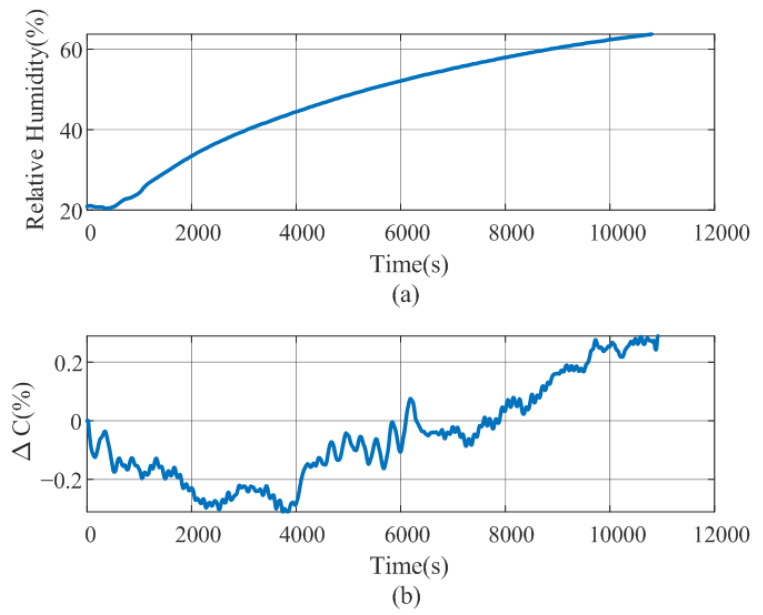
Influence of humidity on the capacitance sensor readings: (**a**) Relative humidity (%) inside the climatic chamber, and (**b**) relative variation of the capacitance as a function of time.

To investigate the influence of ambient humidity, a series of measurements were taken inside a climate chamber (Memmert ICH110L, Bremen, Germany). The setup consisted of a 25 mm diameter glass cylinder and a 1 mm thick absorbent gauze placed on its surface, on which the sensor rested. The assembly was covered with a six-layer bandage to apply a constant pressure equivalent to 40 mmHg. The relative humidity values range from 20% and 80%, following the profile shown in Figure 24a. Figure 24b shows the relative variation of the capacitance as a function of time. The measured capacitance variation remained within ±0.3%. It can be seen that the sensor’s response remains virtually constant until moisture penetrates the bandage. The obtained correlation coefficient was $R^{2}=0.69$. These results indicate that the influence of ambient humidity on the sensor response is limited, since the bandage acts as a protective barrier that reduces moisture absorption and isolates the sensor from direct exposure to humidity variations.

### 4.3. Read Range

**Figure 25:**
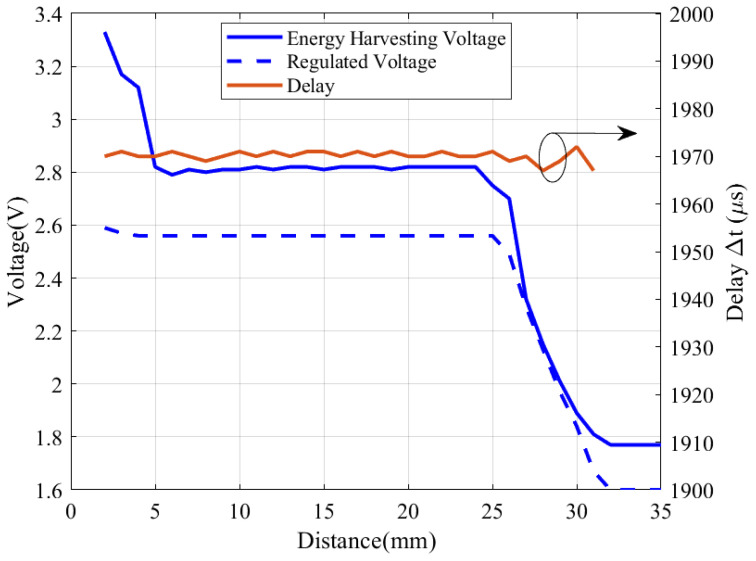
Measured energy-harvesting voltage output and regulated voltage (left axis), and rise time (right axis) as a function of the distance between the NFC board and the smartphone.

The tag has been designed to operate by tapping the mobile device at short range. The output voltage obtained from energy harvesting depends on the distance and coupling between the reader (mobile device) and the NFC coil. Figure 25 shows measurements of both the energy-harvesting output voltage and the regulator output as a function of the distance between the tag and the reader (Xiaomi Redmi 9 smartphone). The distance was controlled using a stepper motor. A capacitor of 150 pF was connected instead of the capacitive sensor to perform these measurements.

The regulator maintains proper operation up to 25 mm, provided that the harvested energy delivers a sufficient input voltage. However, the tag can still operate up to 30 mm as long as the harvested voltage remains above the minimum operating voltage of the microcontroller (1.8 V). The measured delay Δt remains nearly constant, with a deviation of only 0.08%, demonstrating the robustness of the capacitance measurement against variations in the supply voltage.

For larger distances, although the NFC energy-harvesting IC may still provide up to 1.6 V, this voltage is insufficient to sustain operation. As a result, the microcontroller turns off and the tag ceases to respond.

The read range obtained agrees with the expected operating range. When the sizes of the tag and reader coils are comparable, the magnetic coupling coefficient decreases significantly for distances larger than the coil diameter (25 mm in our case), and the harvested energy is no longer sufficient to ensure NFC IC operation [22].

### 4.4. Comparison with Previous Works

Wound monitoring using passive chipless tags based on LC sensors has been extensively investigated in the literature. Table 3 compares the performance of several previously reported LC sensors for pressure or humidity monitoring.

Typically, pressure-sensitive LC sensors employ a flexible spacer material sandwiched between two conductive layers. Applied pressure induces a deformation of the spacer, leading to a change in capacitance and, consequently, a shift in the resonance frequency. An example can be found in [12], where a micromachined silicon diaphragm is used as the sensing element. Another approach proposed in [13] relies on a double-sided structure, in which one side incorporates a printed inductor and the opposite side an interdigitated capacitor, with both elements separated by a layer of poly-L-lactide nanofibers.

In contrast, the sensor developed by the authors in [15] is capable of simultaneously measuring both pressure and moisture levels using a single-layer inductor. The novelty of the proposed approach lies in estimating the moisture level from variations in the resonator quality factor. The high sensitivity achieved by this design is comparable to that reported in [67].

Other works such as [14] have investigated wound healing by analyzing variations in the resonance frequency and the magnitude of the measured reflection coefficient. However, this sensor is not capable of directly measuring pressure. In [67], a dual-frequency LC sensor based on a double-layer structure incorporating a flexible spacer was presented. This design enables simultaneous sensing of pressure and humidity through two distinct resonant modes. Nevertheless, the study only presents preliminary results obtained under controlled pressure chamber conditions and lacks validation on real skin or tissue phantoms.

In addition to calibration challenges and variations in the readout caused by changes in magnetic coupling due to misalignment between the test coil and LC sensors, LC-based systems typically require a vector network analyzer (VNA). Although the operating frequency range allows the use of low-cost VNAs, these instruments must be carefully calibrated using standard loads to achieve high measurement precision, which can complicate their use for non-expert users.

In contrast, batteryless NFC-based sensors require only an NFC-enabled smartphone as a reader, eliminating the need for specialized instrumentation. This ease of use has significantly contributed to the growing adoption of NFC-based solutions for posture and wound monitoring applications. Table 4 summarizes several batteryless NFC-based sensor designs reported in the literature for these use cases.

In [68], a batteryless NFC sensor for strain and temperature sensing based on the RF430FRL152H IC (Texas Instruments, Dallas, TX, USA) was presented. Both sensors are based on the conductive polymer poly(3,4-ethylenedioxythiophene):polystyrene sulfonate (PEDOT:PSS). The strain sensor consists of a microfluidic channel filled with PEDOT:PSS embedded in a polydimethylsiloxane (PDMS) substrate. The proposed strain sensor exhibits a high gauge factor (GF) of 12 500. The sensors were applied as smart sensing devices for monitoring breathing activity, and wound status, which is associated with the typical temperature increase during the healing process. However, the proposed sensor cannot directly estimate either the pressure applied to the wound or the moisture level of the wound.

In [21], an NFC-based posture sensor based on the RF430FRL152H IC (Texas Instruments, Dallas, TX, USA) was presented. The sensor employs tri-layer cantilever strain gauges, whose deformation generates four electrical outputs, enabling the discrimination of both the direction and magnitude of the resultant forces. The proposed sensor can measure pressures up to 45 kPa and integrates a temperature sensor based on a negative temperature coefficient (NTC) thermistor to monitor skin irritation. The system was applied to posture monitoring of patients seated in a wheelchair and lying on a bed.

In [69], an NFC-powered platform capable of simultaneously detecting pressure, temperature, and NH_3_ gas was presented. Pressure sensing was achieved using an optoelectronic pressure sensor, while temperature was monitored using a thermistor. The NH_3_ sensor enables the detection of biological contaminants, such as urine and feces, which release ammonia gas, thereby helping to ensure hygiene and prevent bacterial infections.

A complete posture monitoring systems has been presented in [70]. The system allows real-time monitoring of pressure changes associated with different lying postures using an NFC reader multiplexing two antennas located under the mattress. The pressure was measured using a micromachined strain sensor. The systems also include negative temperature coefficient (NTC) thermistor. The sensor was encapsulated in PDMS.

In addition to pressure monitoring (primarily for posture applications), pH sensing has also been proposed for wound monitoring. In [71], a non-invasive, flexible NFC-based pH sensing system for tracking wound healing progression was presented. The sensor consists of a working electrode and a reference electrode (Ag/AgCl), both fabricated on an indium tin oxide (ITO)-coated substrate. The hydrogen-ion-selective membrane on the working electrode is formed via electropolymerization of aniline, resulting in polyaniline (PANI). The system is based on the SL13 IC from AMS (Premstaetten, Austria).

In [72], a skin moisture sensor (BME280, Bosch Sensortec, Reutlingen, Germany) powered by an NFC interface was used to develop a system for monitoring transepidermal water loss and skin wettedness. The system employs an RF430CL330H NFC transponder (Texas Instruments Inc., Dallas, TX, USA), compliant with the ISO/IEC 14443B standard for data communication, together with an MSP430G2553 microcontroller (Texas Instruments Inc., Dallas, TX, USA) for system management. The biasing and power supply are obtained through Schottky diodes used for rectification of the harvested RF energy.

In contrast to previous works, the proposed system enables simultaneous detection of both the applied pressure and the volume of wound exudate. The system is implemented in two separate modules: a sensor patch and an NFC readout board, in order to improve user comfort. The NFC module integrates an NFC IC for energy harvesting and data communication, along with a microcontroller for system management. The flexible sensing patch is completely passive; it contains no integrated electronic components that could be damaged, and is therefore reusable.

The short read range of NFC-based sensors offers greater privacy and ease of use; however, it can also be a limitation for certain applications, such as continuous monitoring (e.g., in continuous body posture monitoring). In scenarios that require longer communication distances, alternative wireless technologies such as Bluetooth can be employed, although these systems rely batteries.

Measurements taken in wound monitoring applications, such as checking or adjusting the pressure exerted by a bandage are generally intermittent. In these cases, data can be easily collected simply by holding an NFC-enabled smartphone up to the sensor.

Battery-free NFC sensors also offer lower cost compared with other wireless or battery-powered wearable systems, mainly due to the reduced number of electronic components, such as batteries and displays. In addition, their passive operation contributes to a more sustainable and environmentally friendly solution.

## 5. Conclusions

This work has presented a battery-less smart bandage that uses Near-Field Communication (NFC) for wireless monitoring of bandage pressure and wound exudate. The proposed system incorporates a pressure-sensitive resistive sensor and a parallel-plate capacitor for moisture detection within a flexible patch, separated by an absorbent material. To ensure reliability in humid environments, the electrodes of the capacitor are protected with a cover that prevents short circuits. This assembly is connected to a commercial NFC-enabled microcontroller, which improves the device’s portability. The applied pressure is estimated from the resistance variation of the sensitive material using a resistive divider read by the microcontroller, while the wound condition is monitored through capacitance changes induced by exudate absorption in the sensing layer. All electronics are wirelessly powered through NFC energy harvesting and can be interrogated directly with a standard mobile phone.

Experimental results confirm that the system can measure pressures up to 140 mmHg, covering the entire therapeutic range required for compression therapy. It is also capable of detecting exudate volumes of up to 200 μL exhibiting a capacitance variation of approximately 15% between dry and saturated states. The digital reading is performed via a custom-developed Android application, which facilitates real-time data logging and continuous patient monitoring.

Although the current prototype demonstrates promising performance, future work will focus on implementing a fully flexible substrate to improve patient comfort, particularly when the NFC board is embedded into the bandage. In addition, long-term stability studies and clinical trials involving a larger number of participants will be necessary to validate the system’s efficacy in real-world care settings.

## Figures and Tables

**Table 1:** Bandage compression pressure ranges, typical applications, and healing mechanisms.

Pressure (mmHg)	Typical Application	Healing Mechanism/Purpose
10–20	Light compression; for fragile skin, prevention of mild edema	Gentle support; prevents fluid accumulation
20–30	Moderate compression; for mild venous insufficiency	Reduces swelling; improves venous return
30–40	Therapeutic compression; for venous leg ulcers	Improves circulation; promotes tissue oxygenation and wound healing
40–50	High compression; for severe venous insufficiency	Strong venous return; reduces venous hypertension and edema
>50	Specialized/intermittent compression; for lymphedema pumps	Intensive fluid removal; only under strict clinical supervision

**Table 2:** Relative permittivity and thickness of the microstrip layers.

Layer	Relative Permittivity $\varepsilon_{r}$	Thickness *h*
Substrate	$\varepsilon_{rs}$ = 3.4	*h* = 0.1 mm
Spacer layer	$\varepsilon_{r1}$ = 2	$h_{s1}$ = 0.04 mm
Absorption layer (dry)	$\varepsilon_{r2,dry}$ = 2	$h_{s2}$ (variable)
Absorption layer (wet)	$\varepsilon_{r2,wet}=1300$	$h_{s2}$ (variable)
Body (top)	$\varepsilon_{r3}$ = 100	W/2

**Table 3:** Comparison between some LC sensors.

Ref.	Sensing Material	Pressure Range	Sensitivity MHz/kPa	Frequency	Moisture Sensor
[11]	Pyramid PDMS	0–26 kPa	2	725–800 MHz	No
[12]	Silicon diaphragm	0–6.6 kPa	0.9	95–103 MHz	No
[13]	Poly-L-lactide nanofibers	0–16 kPa	1.2	11–14 MHz	No
[14]	Polyimide spiral inductor	–	–	50–110 MHz	No
[67]	Pyramid PDMS	0–6.2 kPa	2.9	80–85 MHz 495–555 MHz	Yes
[15]	Pyramid silicone	0–16 kPa	2.34	290–310 MHz	Yes

**Table 4:** Comparison of battery-less NFC sensors.

Ref.	Pressure Sensor	Pressure Range	Moisture Sensor	Application
[68]	PEDOT:PSS resistance change via strain	Gauge factor 12,000% stretching	No	Smart band with temperature sensor
[21]	Cantilever strain gauges	50 kPa	No	Posture monitoring
[69]	Optoelectronic	10 kPa	No	Posture monitoring, $NH_{3}$ sensor, temperature
[70]	Resistive strain	10 kPa	No	Posture monitoring, temperature sensor
[71]	pH sensor	–	No	Wound monitoring
[72]	Moisture sensor	–	Yes	Transepidermal water loss
This work	Carbon nanocomposite	0–18 kPa	Yes	Smart bandage

## Data Availability

The data presented in this study are available on request from the corresponding author.

## Abbreviations

The following abbreviations are used in this manuscript:

ADC	Analog-to-Digital Converter
CDC	Capacitance-to-Digital Converter
FSR	Force Sensing Resistor
GPIO	General Input/Output
IC	Integrated Circuit
IDE	Interdigital Electrode
LC	Inductor-Capacitor (circuit)
NFC	Near-Field Communication
NTC	Negative Temperature Coefficient
PCB	Printed Circuit Board
PDMS	Polydimethylsiloxane
RF	Radio-Frequency
RFID	Radio-Frequency Identification
TEM	Transverse Electromagnetic
UHF	Ultra-High Frequency
VLU	Venous Leg Ulcer
VNA	Vector Network Analyzer
